# Correlation of β2-microglobulin with postoperative delirium and 3-year mortality undergoing knee or hip replacement surgery: a prospective cohort study

**DOI:** 10.3389/fmed.2025.1597764

**Published:** 2025-08-05

**Authors:** Yuanlong Wang, Qian He, Kun Fu, Yanlin Bi, Bin Wang, Wenjie Kong, Shuhui Hua, Jian Kong, Shanling Xu, Hongyan Gong, Jiahan Wang, Chuan Li, Yanan Lin, Xu Lin

**Affiliations:** ^1^Department of Anesthesiology, Qingdao Municipal Hospital, Qingdao, China; ^2^Second Clinical Medical College, Binzhou Medical University, Yantai, China; ^3^Department of Anesthesiology, Haier Group Yingkang Life, Qingdao, China; ^4^Department of Anesthesiology, Weifang Medical University, Weifang, China

**Keywords:** β2-microglobulin, postoperative delirium, mediation analysis, prediction model, mortality rate

## Abstract

**Introduction:**

Postoperative delirium (POD) is a commonly occurring condition in the postoperative period. Therefore, the study intends to investigate the relationship between B_2_M and POD and the effect of B_2_M levels on three-year postoperative mortality in patients with POD.

**Methods:**

Postoperatively, the Confusion Assessment Method (CAM) and the Monumental Delirium Assessment Scale (MDAS) were used to assess the incidence and severity of POD. Preoperative plasma B_2_M levels were measured utilizing a latex-enhanced immunoturbidimetric assay. Total tau protein (T-tau), phosphorylated tau protein (P-tau), and amyloid β plaque 42 (Aβ_42_) were detected in preoperative cerebrospinal fluid (CSF) by enzyme-linked immunosorbent assay. Logistic regression equations were applied to examine the risk factors linked to POD. Patients presenting with POD were grouped according to B_2_M level and followed up for 3 years postoperatively for their survival and Kaplan–Meier survival curves were plotted.

**Results:**

The prevalence of POD was 7.23%. Serum B_2_M levels were higher in POD patients compared to non-POD (NPOD) patients (*p* = 0.01). The results of the logistic regression analysis indicated that B_2_M (*OR* = 1.394, *95% CI* = 1.017–1.910, *p* = 0.002) and T-tau (*OR* = 1.006, *95% CI* = 1.002–1.011, *p =* 0.007) posed a risk for POD. B_2_M and POD were partially associated through the mediation of CSF T-tau (10.0%). The K-M survival curves showed that patients with high B_2_M who developed POD had a higher mortality rate 3 years after surgery (*p* = 0.031).

**Conclusion:**

In summary, B_2_M may be a risk factor for POD, which might be mediated in part by CSF T-tau.

## Introduction

Postoperative delirium (POD) is defined as an acute impairment of attention and cognition (1). POD is very common in the postoperative period and its main clinical manifestations are altered consciousness, inattention, disorganized thinking, and decreased spatial orientation, occurring 1–7 days after surgery, most commonly 1–3 days after surgery (2, 3). POD is associated with prolonged intensive care, hospitalization, long-term cognitive dysfunction, and shorter survival, which not only affects the health of the patients themselves, but also places an enormous burden on their families and society (4). Therefore, identifying the risk factors associated with POD is particularly critical for early detection.

Although the causes of POD are poorly understood, a growing evidence indicates that oxidative stress, neuroinflammation, circadian rhythm or melatonin dysregulation, and cerebrospinal fluid (CSF) biomarkers play a critical role leading to the development of POD (5, 6). Within these mechanisms, the relationship between CSF biomarkers, which include β-amyloid plaques 42 (Aβ_42_), total tau protein (T-tau), as well as phosphorylated tau protein (P-tau), has been extensively studied. The biomarkers of β-amyloid (Aβ) and tau protein associated with POD in the CSF are associated with abnormal neurological function and are core proteins of POD (7, 8). Studies indicate that patients with POD have lower levels of Aβ protein and higher levels of tau protein in preoperative CSF, and that a decreased Aβ/tau ratio in preoperative CSF is associated with the development of POD (9, 10).

β2-microglobulin (B_2_M), which acts as the light chain of the major histocompatibility complex I (11). It is prevalent in nucleated cells (12, 13). As glomerular filtration rate decreases, B_2_M clearance is impaired, resulting in a gradual increase in its level (14). Smith et al. (15) demonstrated that B_2_M impairs learning and memory in mice. It has also been shown in animal studies that B_2_M causes cognitive deficits in mice (16, 17). Clinical studies have demonstrated a strong correlation between changes in the serum concentration of B_2_M and cognitive function in the perioperative period, whose mechanisms remain to be investigated (18, 19). There are currently no systematic studies to elucidate the relationship between B_2_M, POD and CSF biomarkers.

The present study hypothesizes that B_2_M may be associated with POD, and that CSF biomarkers may mediate this effect. The primary objective of this study was to investigate the relationship between B_2_M and CSF biomarkers and whether the relationship between B_2_M and POD is mediated by CSF biomarkers. Meanwhile, this study investigated the effect of B_2_M on 3 years postoperative survival in patients with POD. This cohort study has been reported in line with the STROCSS guidelines (20).

## Methods

### Participants

This prospective cohort study was conducted between November 2020 and December 2021, and all patients were followed up for 3 years postoperatively. This study was performed in accordance with the Declaration of Helsinki and was approved by the Ethics Committee, which was registered on ClinicalTrials.gov. Then written informed consents from all subjects or legal surrogates were obtained.

Inclusion criteria: aged between 40 and 90 years; no gender restriction; ASA class I-II; patients undergoing knee and hip replacement surgeries under combined spinal-epidural anesthesia. Exclusion criteria were also specified as follow: (1) Patients with Minimental State Examination (MMSE) scores less than 24 (21); (2) Central nervous system infection, epilepsy, multiple sclerosis or other major nervous system diseases; (3) Major psychological disorders (such as depression, delirium, etc.); (4) Severe visual and hearing impairment; (5) Abnormal blood coagulation before operation; (6) renal dysfunction.

### Cognitive measurements

Identical measures were utilized in the cohort studies. A neurologist evaluated patients’ preoperative cognitive function a day before surgery using MMSE, and those with MMSE scores <24 were excluded. Delirium was assessed by a trained anesthesiologist using the Confusion Assessment Method (CAM) (22) twice daily at 9.00–10.00 am and 2.00–3.00 pm for 1–7 days after surgery. The diagnostic criteria for POD were as follows: (1) acute onset with a fluctuating course; (2) inattention; (3) disordered thinking, and (4) changes in the level of consciousness (any state of consciousness other than fully conscious). The diagnosis of POD requires the presence of (1) and (2), accompanied by (3) or (4) or both. Patients who developed postoperative delirium (POD) underwent additional evaluation for delirium severity using the Memorial Delirium Assessment Scale (MDAS) (23). Subsequently, patients were divided into POD and non-POD (NPOD) groups based on their development of POD. The three-year postoperative survival of patients with POD was obtained using telephone follow-up.

### Anesthesia and surgery

The study involved patients who had taken no medications preoperatively, fasted for at least 8 h, and abstained from water for at least 4 h (24) Healthcare providers routinely monitor the patient’s vital signs, such as electrocardiogram, pulse oximetry, and non-invasive blood pressure, before administering anesthesia. Peripheral venous access was established. Venous blood samples were collected from 6 am to 7 am on the day of surgery.

The patient received combined lumbar-rigid anesthesia. Firstly, 2 mL of cerebrospinal fluid samples were extracted from the subarachnoid space. Next, 2–2.5 mL of 0.66% ropivacaine was injected. The plane of anesthesia was adjusted at the level of thoracic 8 and oxygen was administered to the patients via a mask at a rate of 5 L/min during the operation. All patients were not sedated intraoperatively. Vasoactive drugs were administered to maintain the stability of vital signs based on the specific conditions of each patient during the operation. After surgery, patients were promptly transferred to the PACU and were closely monitored for 30 min prior to being sent back to the ward. The postoperative analgesic regimen used patient controlled intravenous analgesia (Butorphanol 10 mg + Tolansetron 5 mg + 0.9% saline solution 89 mL) to maintain a postoperative visual analog score (VAS) < 3, with non-steroidal analgesics administered as required.

### Plasma B_2_M determination

Plasma B_2_M levels were measured with a latex-enhanced immunoturbidimetric assay using an automated biochemical analyzer (Beckman Coulter Automated Biochemistry Analyzer AU5800, United States), following standard clinical laboratory procedures. The assay was conducted after a 12-h overnight fast at the Qingdao Municipal Hospital’s clinical chemistry laboratory in China.

### CSF biomarker assessments

The CSF samples were procured from participants and subjected to centrifugation at 2000 g for a duration of 10 min under room temperature. Thereafter, they were preserved at −80°C for supplementary analysis. The concentrations of Aβ_42_, T-tau and P-tau in the CSF were measured via enzyme-linked immunosorbent assay (ELISA) utilizing an enzyme labeling instrument (Thermo Scientific Multiskan MK3). The concentrations of Aβ_42_/T-tau and Aβ_42_/P-tau were calculated.

### Sample size estimation

Following initial research, the study intended to include eight covariates. In addition, the incidence of POD was established to be 11% based on preliminary findings. It was assumed that the rate of patient loss to follow-up during the postoperative period would be 20%. Thus, the necessary sample size of the study required 910 cases [8 × 10 ÷ 11% ÷ (1–20%) = 910].

### Statistical analysis

Participant characteristics are presented as either mean with standard deviation (SD), median and interquartile range (IQR 25th-75th percentile), or as a percentage (%). To test for normality, the Kolmogorov–Smirnov test was used for all variables. When data followed a normal distribution, independent samples t-tests were used to compare group differences. For continuous variables that were non-normally distributed, non-parametric methods were employed. The Mann–Whitney *U* test was utilized to compare inter-group differences, while the *χ^2^* test was employed to compare categorical variables. Incidence of POD was denoted as a percentage.

The variables that were significant were included in univariate regression analyses and then multivariate logistic regression analyses were conducted after adjustment for ASA classification and sex. Two sensitivity analyses were then conducted. The first Adjusted for age, ASA, gender, years of education, BMI, smoking history, drinking history, MMSE scores, Hypertension, Diabetes, coronary heart disease to the multivariate logistic regression analysis, and the second added Duration of surgery, Duration of anesthesia, Intraoperative fluid, Estimated blood loss to the first. Linear regression analysis was utilized to correlate B_2_M levels with CSF biomarkers.

In order to investigate whether the relationship between the B_2_M and the POD was influenced by the presence or absence of POD pathology, we carried out a mediation analysis using the approach suggested by Baron and Kenny (25). To establish mediation, four key criteria had to be met simultaneously: (a) inclusion of the mediator (CSF biomarker) in the regression model attenuated the association between B_2_M and POD; (b) a notable correlation between CSF biomarker and POD was observed; (c) the correlation between B_2_M and POD was apparent; (d) a significant correlation between B_2_M and CSF biomarker existed.

The dynamic nomograms was used to determine the occurrence of POD in combination with other influencing factors (Age, Education, MMSE, ASA grade). This approach was used to substantiate the status of B_2_M as a risk factor for POD and to construct a corresponding clinical prediction model. Meanwhile, we plotted the ROC curve and assessed the diagnostic effect of the model by AUC. To assess calibration, a logistic model was fitted, and the fitted model provided the estimated probability of an observation conditional on the predicted risk. Observed and predicted risks were plotted against each other to generate flexible calibration curves. In addition, we utilize clinical decision curve (DCA) to evaluate the net benefit that the model provides to patients by influencing clinical decisions. Time-to-event results were analyzed with Kaplan–Meier survival analyses with log-rank tests.

In a *post hoc* analysis, we replicated model 2b for the study and excluded patients with MMSE <28, education <9, and diabetes to assess whether our conclusions held true in a cognitively better, more educated, and non-diabetic population.

A statistically significant difference was defined as *p* < 0.05. All statistical techniques and graphs above were generated using SPSS (version 25.0), R (version 4.3.1), GraphPad Prism (version 9.4.2), and Stata (version 15.1).

## Results

### Patient characteristics

**Figure 1:**
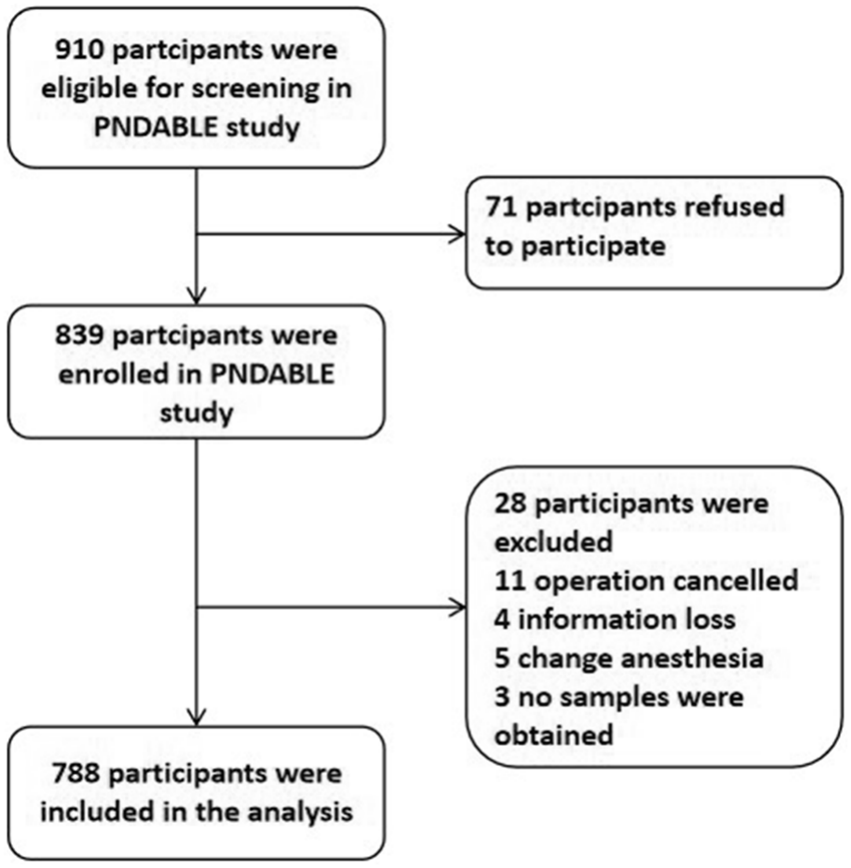
Flow diagram of the perioperative neurocognitive disorder and biomarker lifestyle (PNDABLE) study.

We enrolled a total of 910 participants, of whom 788 were eligible for this study and 122 were excluded. Figure 1 presents the reasons. Fifty-seven enrolled patients developed POD within 7 days postoperatively or before discharge. Demographic and clinical data for the study participants is also displayed in Table 1. The incidence of POD was 7.23%. Significant differences were found between POD and NPOD groups in terms of age, gender, education, hypertension, MDAS, ASA grade, and intraoperative fluid as determined via Mann–Whitney U test (*p* < 0.05). Additionally, there were significant differences in CSF Aβ_42_, T-tau, Aβ_42_/T-tau, Aβ_42_/P-tau between the two groups (*p* < 0.05).

**Table 1:** Demographic and clinical characteristics of participants in the perioperative neurocognitive disorder and biomarker lifestyle (PNDABLE) study.

Characteristic	POD (*n* = 57)	NPOD (*n* = 731)	*P*
Age [year, *M*(IQR)]	73(70–76)	63(56–68)	<0.001^***^
Male [*n*(%)]	39(68.42)	399(54.58)	0.043*
Education [year, *M*(IQR)]	7(6–9)	10(9–12)	<0.001***
Height [cm, *M*(IQR)]	165(160–170)	167(160–172)	0.535
Weight [kg, *M*(IQR)]	69(61.5–76.5)	70(62–78)	0.372
BMI [kg/m^2^, *M*(IQR)]	24.77(22.82–28.08)	25.39(23.26–27.64)	0.357
MMSE [scores, *M*(IQR)]	29(27.5–30)	28(27–29)	0.094
Smoking history [*n*(%)]	14(24.56)	185(57.63)	0.901
Drinking history [*n*(%)]	14	228	0.296
Hypertension [*n*(%)]	31	269	0.008**
Diabetes [*n*(%)]	15	132	0.123
CHD [*n*(%)]	7	66	0.415
β2-microglobulin [mg/L, M(IQR)]	1.89(1.51–2.47)	1.67(1.35–2.06)	0.010*
MDAS [scores, M(IQR)]	13(12–18.5)	1(1–5)	<0.001**
ASA grade [*n*(%)]			0.036*
I	1(1.75)	75(10.26)	
II	56(98.25)	656(89.74)	
Duration of surgery [h, M(IQR)]	1.58(0.83–2.17)	1.58(1.00–2.08)	0.411
Duration of anesthesia [h, M(IQR)]	2.25(1.38–2.92)	2.42(1.75–3.00)	0.208
Intraoperative fluid [ml, M(IQR)]	1,100(600–1,150)	1,100(1000–1,500)	0.046*
Estimated blood loss [ml, M(IQR)]	20(5–200)	30(10–200)	0.635
VAS 24 h postoperatively [scores, M(IQR)]	1(0–2)	1(0–2)	0.854
Aβ_42_ [pg/ml, M(IQR)]	335.53(231.71–400.61)	425.52(285.70–598.90)	<0.001***
T-tau [pg/ml, M(IQR)]	205.93(145.95–306.99)	180.20(140.06–226.00)	0.006*
P-tau [pg/ml, M(IQR)]	42.20(33.44–52.58)	39.68(32.07–48.26)	0.083
Aβ_42_/T-tau[M(IQR)]	1.63(0.93–2.33)	2.37(1.52–3.51)	<0.001**
Aβ_42_/P-tau[M(IQR)]	8.00(5.03–10.78)	10.90(7.21–15.38)	<0.001**

CHD, coronary heart disease; MMSE, Mini-Mental State Examination; POD, postoperative delirium; VAS, visual analog score.

*P-value < 0.05.

**P-value < 0.01.

***P-value < 0.001.

### The correlation of B_2_M with POD

Multifactorial regression analysis adjusted for confounders showed that B_2_M (*OR* = 1.394, *95% CI* = 1.017–1.910, *p* = 0.002) remained a risk factor for POD, as shown in Table 2. We also performed a two-step sensitivity analysis. Both sensitivity analysis steps were statistically significant.

**Table 2:** Logistic regression on analysis and sensitivity analysis.

	Model 1^a^		Model 2^b^		Model 3^c^		Model 4^d^	
	OR (95% CI)	*P*	OR (95% CI)	*P*	OR (95% CI)	*P*	OR (95% CI)	*P*
β2-microglobulin mg/L	1.565(1.181–2.073)	0.002*	1.394(1.017–1.910)	0.039*	1.524(1.116–2.082)	0.008*	1.457(1.016–2.090)	0.041*
Aβ_42_ pg./ml	0.996(0.994–0.998)	<0.001*	0.993(0.989–0.997)	0.002*	0.996(0.994–0.998)	<0.001*	0.994(0.991–0.997)	<0.001*
T-tau pg./ml	1.006(1.003–1.009)	<0.001*	1.006(1.002–1.011)	0.007*	1.005(1.002–1.008)	0.004*	1.005(1.000–1.009)	0.029*
P-tau pg./ml	1.024(1.004–1.044)	0.019*	1.038(1.001–1.077)	0.043*	1.016(0.995–1.038)	0.133	1.019(0.994–1.045)	0.141
Aβ42/T-tau	0.515(0.386–0.686)	<0.001*	1.274(0.830–1.956)	0.269	0.518(0.382–0.701)	<0.001*	0.474(0.326–0.689)	<0.001*
Aβ42/P-tau	0.850(0.793–0.911)	<0.001*	1.053(0.906–1.224)	0.502	0.843(0.782–0.910)	<0.001*	0.793(0.715–0.881)	<0.001*

OR, odds ratio; 95% CI, 95% confidence interval.

^a^Model 1: Unadjusted.

^b^Model 2:adjusted for ASA and gender.

^c^Model 3: Adjusted for age (50–90 years), ASA, gender, years of education, BMI, smoking history, drinking history, MMSE scores, hypertension, diabetes, and coronary heart disease.

^d^Model 4: Adjusted for age (>65 years), ASA, gender, years of education, BMI, smoking history, drinking history, MMSE scores, hypertension, diabetes, coronary heart disease, duration of surgery, duration of anesthesia, intraoperative fluid, and estimated blood loss.

*p < 0.05.

### Relationship between CSF biomarkers and POD

We compared the concentrations of the preoperative CSF biomarkers Aβ_42_, T-tau, and P-tau, as well as Aβ_42_/T-tau and Aβ_42_/P-tau in both POD and NPOD patients in addition to B_2_M. The Mann–Whitney test indicated significantly higher cerebrospinal fluid T-tau levels in POD patients compared to NPOD patients. In addition, CSF Aβ_42_ levels were significantly lower in POD patients than in NPOD patients. Unadjusted regression analysis indicated that all of the above CSF biomarkers were statistically significant; however, when adjusted multivariate regression was performed, Aβ_42_/T-tau (*OR* = 1.274, *95% CI* = 0.830–1.956, *p* = 0.269) and Aβ_42_/P-tau (*OR* = 1.053, *95% CI* = 0.906–1.224, *p* = 0.502) were not statistically significant. Nevertheless, T-tau (*OR* = 1.006, *95% CI* = 1.002–1.011, *p =* 0.007), P-tau (*OR* = 1.038, *95% CI* = 1.001–1.077, *p* = 0.043) and A*β*_42_ (*OR* = 0.993, *95% CI* = 0.989–0.997, *p* = 0.002) remained statistically significant. Two rounds of sensitivity analysis showed that T-tau was a risk factor for POD, A*β*_42_ was a protective factor for POD, while P-tau was not statistically significant (Table 2).

### The correlation of B_2_M with CSF biomarkers

**Figure 2:**
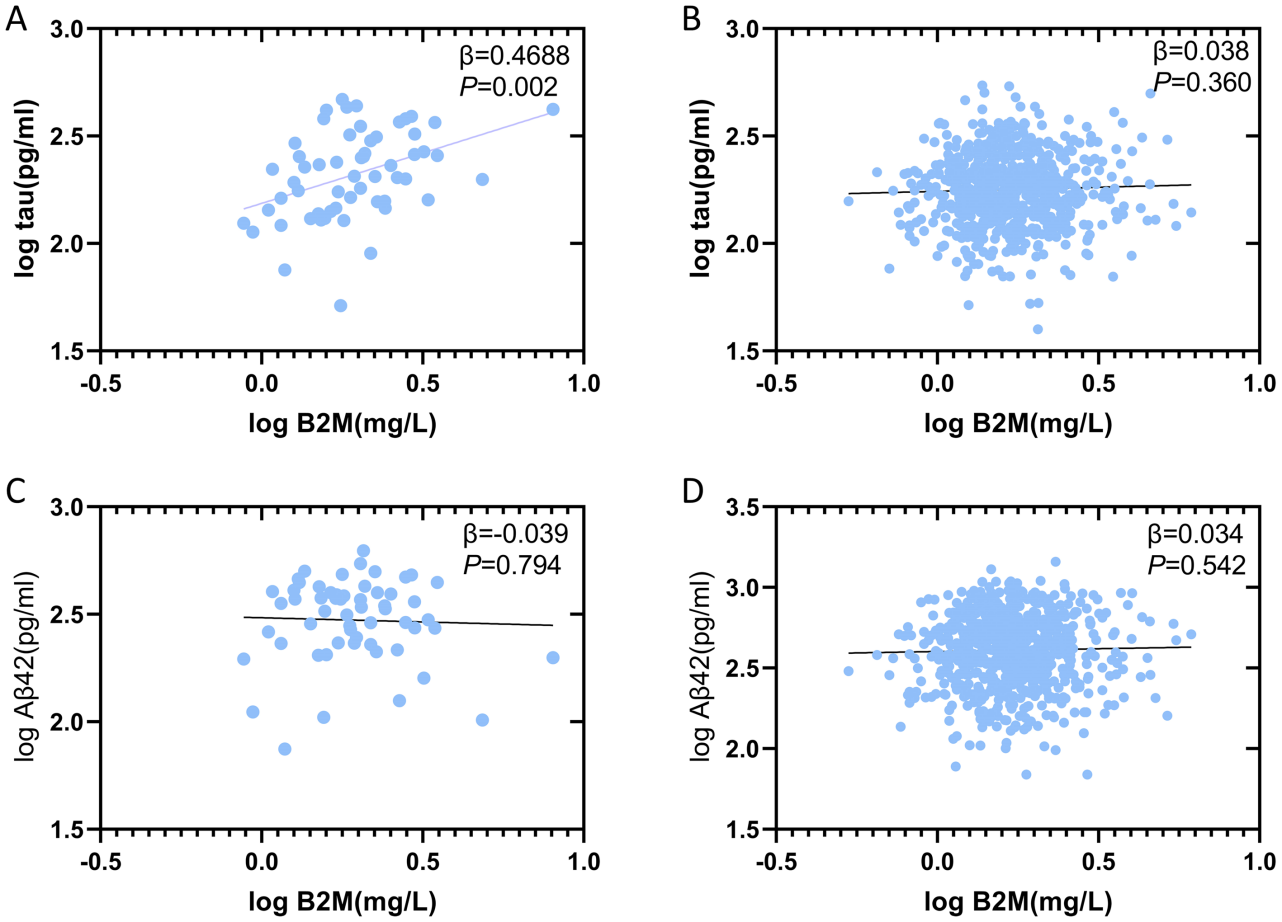
Linear regression of B_2_M with CSF biomarkers. Scatterplot representation of the relationship between B_2_M and CSF biomarkers: relationship between Aβ42, T-tau in different groups [POD **(A,C)**, NPOD **(B,D)**]. CSF, cerebrospinal fluid; B_2_M, β2-microglobulin; Aβ42, amyloid-β42; T-tau, total Tau.

Multiple linear regression analysis revealed a significant positive correlation (*β* = 0.469, *p* = 0.002) between B_2_M and CSF T-tau in POD patients, whereas no such correlation (*β* = 0.038, *p* = 0.360) was observed in NPOD patients. Aβ_42_ did not demonstrate the same relationship (Figure 2).

### Mediation analyses

**Figure 3:**
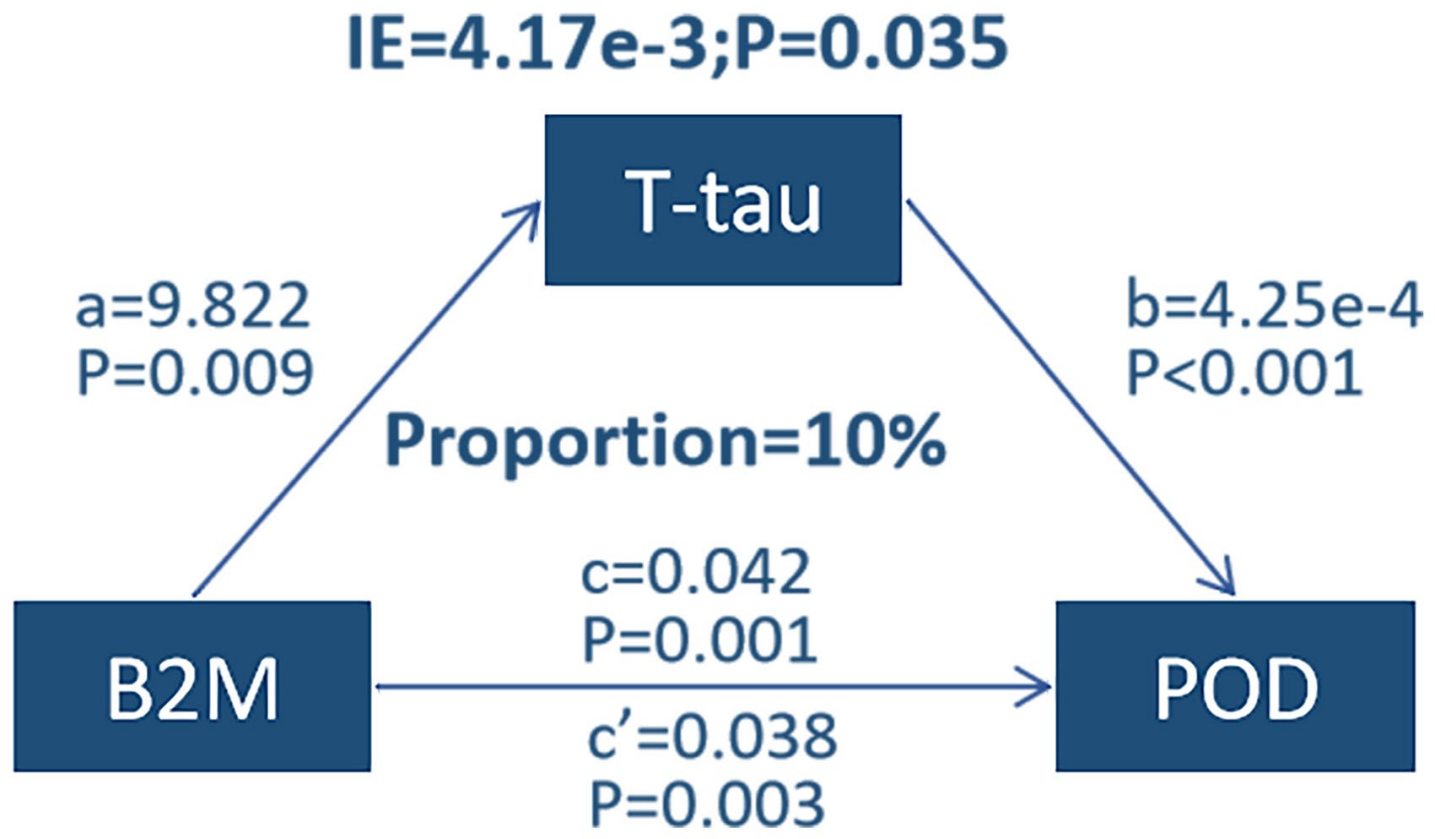
Mediation analyses.

Mediation analyses revealed that the relationship between B_2_M and POD was partially mediated by a mediating effect of T-tau, with a proportion of mediators of approximately 10% (Figure 3).

### Predictive model

**Figure 4:**
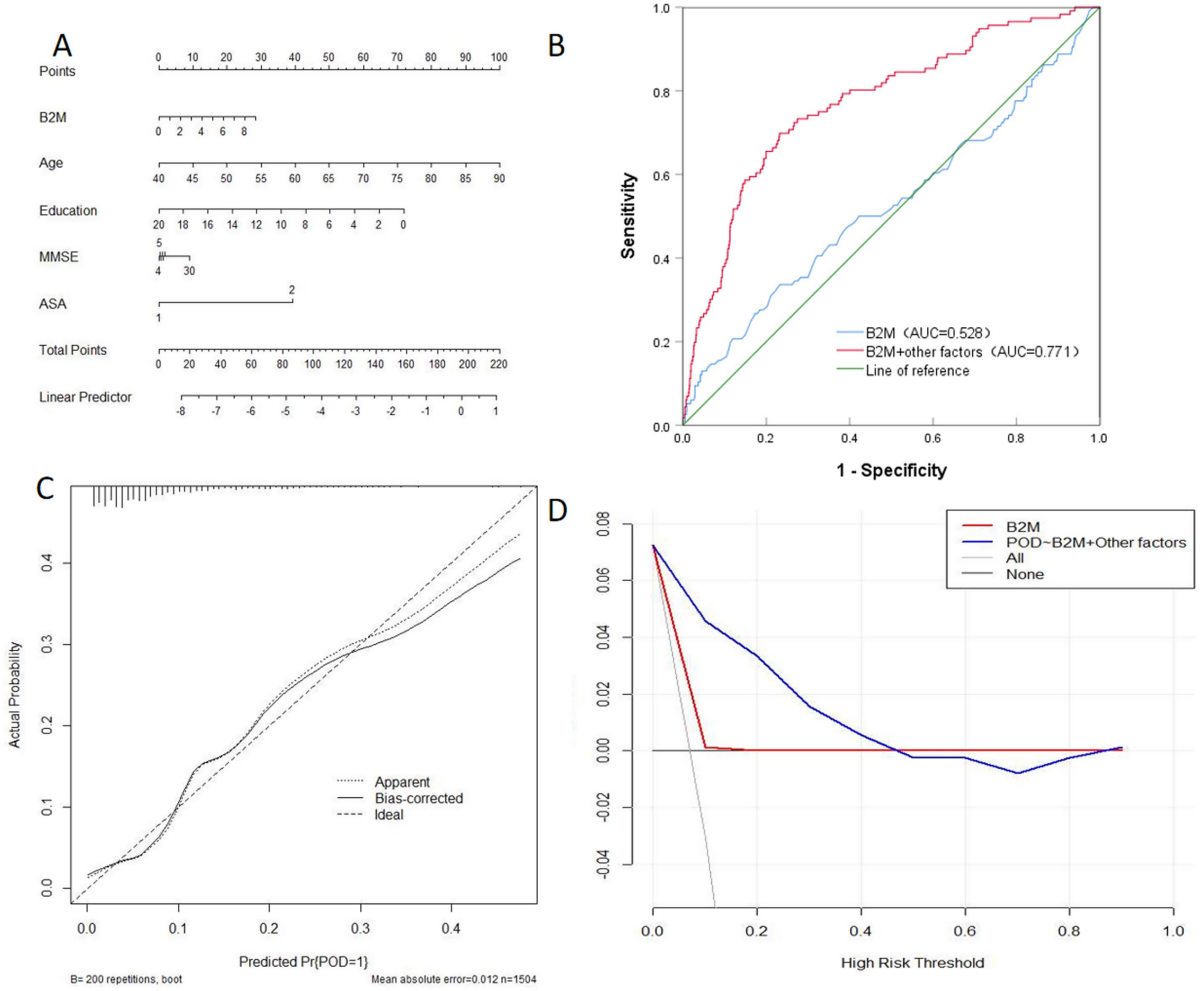
Dynamic nomogram **(A)**, ROC curves **(B)**, calibration curves **(C)** and decision curves **(D)**. B_2_M, β2-microglobulin. Other factors: age, education, MMSE, ASA grade.

The study showcased the predictive potential of B_2_M and the associated metrics (age, education, MMSE score, ASA grade) for POD through a Dynamic Nomogram. Results from the ROC curve strongly suggested that B_2_M and its associated metrics (AUC = 0.771) provided more precise predictions for POD than B_2_M alone (AUC = 0.528). Further supporting the predictive ability of the model, the calibration curve and DCA curves also displayed the effectiveness of the Dynamic Nomogram (Figure 4).

### Kaplan–Meier survival analyses

**Figure 5:**
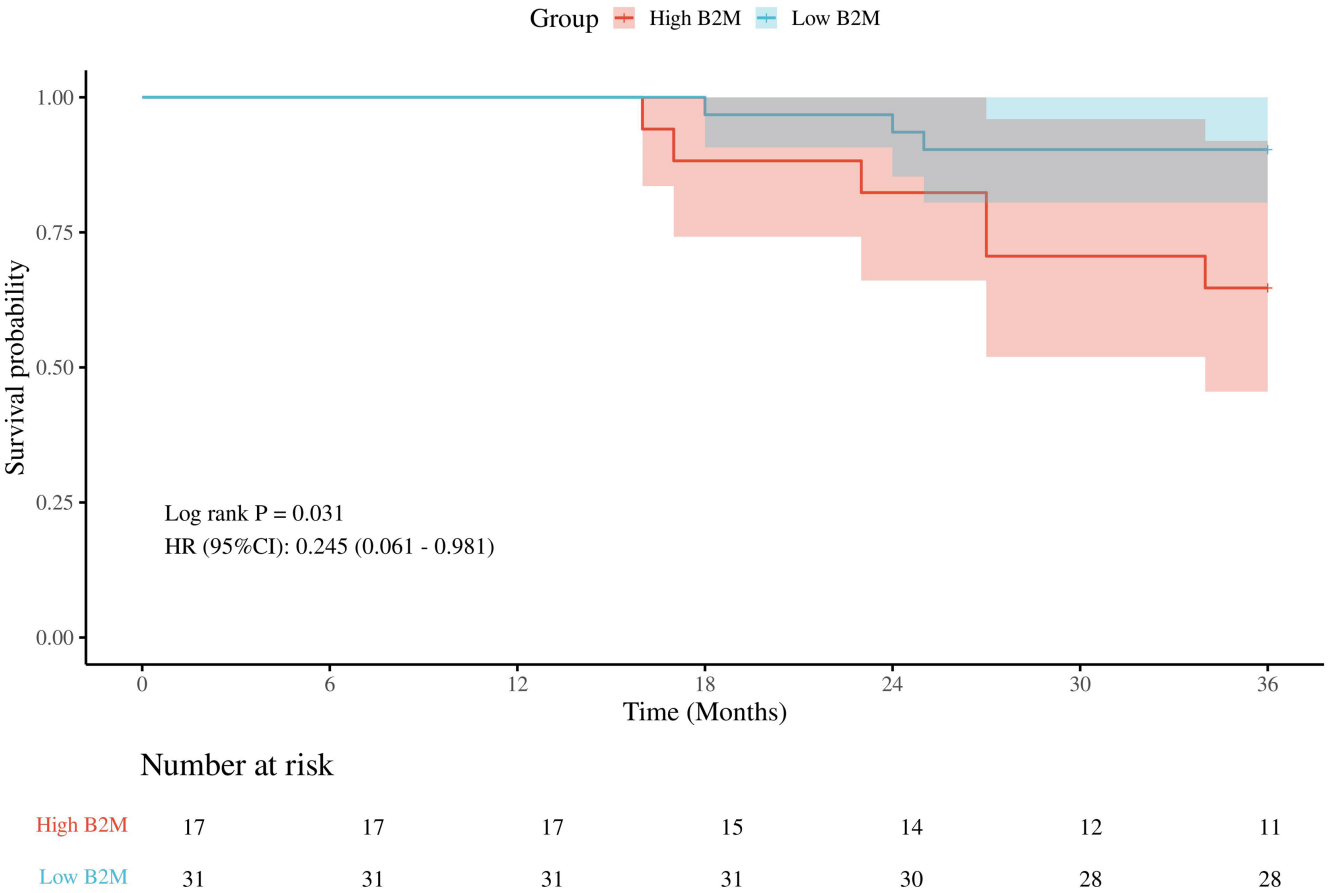
The Kaplan–Meier survival analysis.

There were a total of 57 patients with POD, of which 9 patients were lost to follow-up. There were 17 patients with high B_2_M and 6 (35.3%) deaths; 31 patients with low B_2_M and 3 (9.7%) deaths. Classification of POD patients into low B_2_M and high B_2_M groups based on 75% quartiles (2.1 mg/L). The Kaplan–Meier survival analysis demonstrated that the three-year postoperative survival rate was significantly lower in the high B_2_M group than in the low B_2_M group (*p* = 0.031), in Figure 5.

### *Post hoc* analyses

We performed a replication of the study by excluding individuals with MMSE < 28, education level < 9, and diabetes. The results are respectively: Model 1 [B_2_M (*OR* = 1.460, *95% CI* = 1.038–2.052, *p* = 0.029)]; Model 2 [B_2_M (*OR* = 1.577, *95% CI* = 1.077–2.311, *p* = 0.019)]; Model3 [B_2_M (*OR* = 1.520, *95% CI* = 1.064–2.170, *p* = 0.021)]. The results demonstrated that these exclusions had no impact on our findings, which reveals that B_2_M is a risk factor for POD (Table 3).

**Table 3:** *Post hoc* analyses.

	Model 1^a^		Model 2^b^		Model 3^c^	
	OR (95% CI)	*P*	OR (95% CI)	*P*	OR (95% CI)	*P*
β2-microglobulin mg/L	1.460(1.038–2.052)	0.029*	1.577(1.077–2.311)	0.019*	1.520(1.064–2.170)	0.021*
Aβ_42_ pg./ml	0.990(0.984–0.995)	<0.001*	0.994(0.986–1.001)	0.111	0.992(0.987–0.997)	0.002*
T-tau pg./ml	1.007(1.001–1.013)	0.016*	1.008(1.001–1.014)	0.022*	1.006(1.001–1.011)	0.014*
P-tau pg./ml	1.071(1.030–1.114)	0.001*	1.025(0.961–1.093)	0.458	1.049(1.006–1.094)	0.025*
Aβ42/T-tau	1.435(0.820–2.512)	0.206	1.471(0.875–2.473)	0.145	1.347(0.882–2.056)	0.168
Aβ42/P-tau	1.133(0.970–1.323)	0.115	0.973(0.726–1.306)	0.857	1.067(0.897–1.270)	0.462

OR, odds ratio; 95% CI, 95% confidence interval.

^a^Model 1: Exclude MMSE < 28.

^b^Model 2: Exclude Education < 9.

^c^Model 3: Exclude Diabetes.

*p < 0.05.

## Discussion

Our study demonstrated that B_2_M may be a risk factor for POD, and CSF T-tau partially mediates this effect. In addition, it was found that patients with high B_2_M who developed POD had a higher three-year postoperative mortality rate. The model we developed by combining B_2_M and other relevant indicators effectively predicts POD, allowing for early detection and prevention, and ultimately reducing its incidence.

Our study concluded that B_2_M may be a risk factor for POD, which is consistent with the previous research linking B_2_M to cognitive impairment (17, 18). We strengthened the validity of our findings with a sensitivity analysis and a *post hoc* analysis. At the same time, T-tau as a risk factor for POD and Aβ_42_ as a protective factor for POD were also demonstrated in our study. Several mechanisms by which B_2_M causes cognitive dysfunction have been uncovered in the past. B_2_M is the light chain of MHC-I, with regulatory effects on brain development and synaptic plasticity, and is thought to contribute to cognitive deficits (26). Furthermore, the research has revealed that B_2_M has the capability to impact the characteristics of hippocampal neural progenitor cells (NPCs) potentially leading to cognitive dysfunction (27, 28). Examine has demonstrated the presence of immune responses (involving B_2_M) in the brain, which may lead to neuronal damage (29, 30). In conclusion, B_2_M may lead to cognitive dysfunction through its impact on NPCs activity, immune response, and related pathways. However, the mechanism that is responsible for B_2_M-induced POD remains unexplored.

Plasma B_2_M correlates with CSF biomarkers, including Aβ and tau proteins, which may play an important role in B_2_M’s association with cognitive impairment (31). Our results indicate that CSF T-tau levels were significantly higher in patients with POD compared to NPOD, whereas Aβ_42_ levels were significantly lower in POD patients compared to NPOD patients. Additionally, only in the POD cohort did we find a significant correlation between T-tau and B_2_M, while no such association between Aβ_42_ and B_2_M was found in both groups. We have found a significant correlation between B_2_M and POD, as well as a clear correlation between B_2_M and CSF T-tau. Therefore, we hypothesized that the correlation between B_2_M and POD may be mediated by T-tau.

Next, the mediating effect was implemented to substantiate our hypothesis. The association between B_2_M and POD was partly moderated by the mediating influence of T-tau (10%), while P-tau did not play a role as a risk factor for POD. This suggests that the effect of B_2_M on POD may be partially influenced by the blocking of tau protein. However, it is unclear how B_2_M facilitates T-tau in causing POD. Tau protein is abundant in axonal compartments of neurons and also presents at lower levels in oligodendrocytes and astrocytes (2). Its main functions include regulating microtubule assembly, nucleation, and bundling, as well as the regulation of axonal transport (32). In sum, additional research is necessary to determine the mechanism through which B_2_M facilitates T-tau, and POD subsequently. Currently, multiple studies have demonstrated that P-tau can induce pathological changes through a variety of mechanisms (33–35). Except phosphorylation, tau protein undergoes a variety of other post-translational modifications, such as acetylation, glycosylation (36), saccharification, deamidation, isomerization, nitration, methylation, ubiquitination, sumoylation, and truncation (37). Consequently, we hypothesized that the effect of B_2_M on POD may be mediated by tau proteins other than P-tau, which remains to be investigated in the future.

For patients diagnosed with chronic kidney disease, the management of hypertension, diabetes and other diseases that result in renal injury, followed by the enhancement of renal function, has been identified as a efficacious approach to reduce B_2_M (38). Moreover, for patients with end-stage renal disease, high-throughput hemodialysis or hemofiltration has been shown to be a more efficient method for the elimination of B_2_M (39). It has been demonstrated that immunotherapy (e.g., anti-tau antibodies) (40) or autophagy activators (e.g., rapamycin) (41) may accelerate aberrant degradation of tau proteins. These measures have the potential to reduce the occurrence of POD in patients and improve survival by lowering B_2_M or tau protein, but further validation through clinical trials is required to substantiate these findings.

The findings of this study indicated a substantially elevated three-year postoperative mortality rate in patients with elevated B_2_M levels on POD. Concurrent studies have established a correlation between B_2_M and arterial disease, as well as vascular structural changes, and have identified an association with inflammatory responses (42). Furthermore, B_2_M has been demonstrated to induce smooth muscle cell vitrification, which can subsequently result in atherosclerosis by means of a mediation process involving inflammatory response factors, including soluble viscous molecules. (43) In addition, the chemotactic effect of B_2_M on thrombogenic mononuclear macrophages accelerates thrombosis and is involved in the onset and development of atherosclerosis (44). It has been demonstrated that in patients with CKD, a 1 mg/L increase in serum B_2_M levels is associated with an 18% increased risk of all-cause mortality and a 22% increased risk of cardiovascular death (45). Another study has shown that elevated B_2_M levels are significantly associated with short-term mortality in patients with acute heart failure (46). In conclusion, elevated B_2_M levels have the potential to influence mortality through multi-organ interactions, involving the kidneys, cardiovascular system, immune system, and nervous system. Further research is required to elucidate the pathological mechanisms of B_2_M elevation using multi-omics analysis and other methodologies.

It is worth noting that microglia B_2_M may alter astrocyte function and phenotype, further affecting the blood–brain barrier (BBB), Central Nervous System (CNS) immune homeostasis, synaptic plasticity, and conventional neuronal communication (47). Reactive astrocytes overexpress glial fibrillary acidic protein (GFAP), which has also been found to play a role in the pathophysiology of tau proteins and amyloid in the brain (48). In addition, B_2_M not only acts as a component of GFAP (49). When MHC-I is destabilized, high levels of B_2_M overactivated reactive astrocytes, leading to astrocyte proliferation and elevated GFAP levels, which can lead to neuronal dysfunction and increased neurotoxicity, which can further affect tau protein pathology (50). Knockdown of MHC-I expression reduces astrocyte proliferation, whereas B_2_M silencing leads to astrocyte atrophy by reducing GFAP expression (51).

In this study, mediation analyses were performed to examine how CSF biomarkers mediate the correlation between B_2_M and POD. Several factors improved the reliability of the study. To begin with, we performed a variety of data validations, including sensitivity analyses and *post hoc* analyses. In addition, we developed a clinical prediction model that proved to be an important tool for predicting POD in patients and taking preventive measures in advance. In this study, nomograms were produced to facilitate clinical application, which can be used to predict POD risk by relevant indicators in patients before surgery. This facilitates preoperative risk assessment, improved intraoperative management of patients at high risk of POD, and early postoperative intervention. Finally, we followed POD patients for survival 3 years after surgery, which makes the study more valuable in the long term.

However, our study has limitations. Firstly, this study only investigated the correlation between B_2_M and POD, as well as the mediating role of CSF biomarkers. However, it would be beneficial for future research to explore other potential mechanisms of association between B_2_M and POD. Secondly, the study was a single-center design, which limits its generalisability. Consequently, the results of this study require greater generalisability, which can be achieved through prospective multicenter studies. In addition, the predictive model needs to be validated externally in order to test it. Furthermore, the incidence of POD in this study was lower than the 11% assumed in the sample size calculation, which may have a bearing on the reliability of the conclusions. Next, in this study, methods such as propensity score matching were not employed to balance the variables between the POD and non-POD groups. However, multivariate logistic regression was utilized to compensate for the potential limitations of not using propensity score matching. In future studies, we will apply the propensity score matching method by developing better inclusion and exclusion criteria, lowering the loss-to-follow-up rate, and increasing the sample size in order to validate the current findings. Finally, it is acknowledged that the study may have been influenced by a number of potential confounders. In subsequent studies, the potential for bias reduction may be enhanced by the inclusion of a more extensive and heterogeneous group of participants.

## Conclusion

In summary, B_2_M may be a risk factor for POD, which might be mediated in part by CSF T-tau. The prediction model we constructed by combining B_2_M and other related indicators can effectively predict the occurrence of POD, which will be helpful for us to detect and prevent POD in advance and thus reduce the occurrence of POD.

## Data Availability

The raw data supporting the conclusions of this article will be made available by the authors, without undue reservation.
